# Metabolic profiling of therapy-induced senescent cancer cells via TPEF, MALDI-MS, and RNA-sequencing

**DOI:** 10.1038/s41598-025-32573-y

**Published:** 2025-12-17

**Authors:** Silvia Ghislanzoni, Federica Padelli, Matteo Niero, Alessia Bertolotti, Antonino Belfiore, Simone Torelli, Arianna Bresci, Andrea Masella, Silvia Betti, Dario Polli, Luca Agnelli, Italia Bongarzone

**Affiliations:** 1 Department of Diagnostic Innovation, Fondazione IRCCS Istituto Nazionale dei Tumori, Via Giacomo Venezian 1, Milan, 20133 Italy; 2 Physics Department, Politecnico di Milano, Piazza L. da Vinci 32, 20133 Milano, Italy; 3 G. R. Harrison Spectroscopy Laboratory, Massachusetts Institute of Technology, Cambridge, MA 02139 USA; 4 Datrix S.p.A, Foro Buonaparte 71, Milan, 20121 Italy; 5 CNR Institute for Photonics and Nanotechnologies (IFN), Piazza L. da Vinci 32, Milan, 20133 Italy

**Keywords:** Senescence, Breast cancer, Engulfing, Biochemistry, Cancer, Cell biology, Molecular biology

## Abstract

Despite advances in cancer therapies, treatment failure from resistance and recurrence remains a major clinical challenge. Therapy-induced senescence (TIS), a state of stable cell cycle arrest with sustained metabolic activity, has emerged as a driver of inflammation, tumor persistence, and relapse. However, the heterogeneity of TIS complicates its detection and targeting. Here, we applied a multi-modal strategy to characterize metabolic alterations in senescent cancer cells induced by doxorubicin or γ-irradiation across three tumor cell lines: MCF7, HeLa, and TPC-1. Mitochondrial dysfunction was assessed using MitoTracker and JC-1 staining, while two-photon excitation fluorescence (TPEF) microscopy enabled label-free visualization of intracellular NAD(P)H and FAD distribution. Lipid remodeling was evaluated by MALDI mass spectrometry imaging, and RNA sequencing was performed on control, senescent, and engulfing-senescent MCF7 cells to identify differentially expressed genes and enriched pathways. Senescent cells displayed mitochondrial dysfunction, with altered NAD(P)H/FAD distribution and decreased membrane potential. TPEF confirmed redistribution of coenzymes, reflecting redox changes. Lipidomics revealed consistent remodeling, notably involving cardiolipin precursors. Transcriptomic profiling showed engulfing-senescent MCF7 cells possess a distinct signature marked by increased lipid metabolism, endocrine signaling, and cell–cell communication. Overall, our findings reveal conserved and cell type–specific metabolic traits of TIS, highlighting metabolic vulnerabilities for senolytic intervention.

**Supplementary Information:**

The online version contains supplementary material available at 10.1038/s41598-025-32573-y.

## Introduction

In response to anti-tumor therapy, a subset of cancer cells avoids apoptosis by entering a state known as senescence, characterized by stable cell cycle arrest, extensive macromolecular remodeling, and a hypersecretory, pro-inflammatory phenotype^1,2^. It is now recognized how senescence plays a dual role in the cancer development^3,4^: on the one hand, the persistent cytostatic state of senescent cells might act as a natural barrier against cancer growth^2,5^. On the other hand, the inflammatory factors secreted by long term senescent cells promote survival, proliferation, and stemness, modulating their microenvironment toward a pro-carcinogenic type^4^. Understanding this balance is critical to exploit senescence as an anti-cancer strategy or selectively target senescent cells to mitigate their detrimental effects. Yet, the lack of clear, specific criteria for recognizing senescent cells critically limits these efforts. Senescence is a highly complex, dynamic phenomenon characterized by heterogeneous phenotypes^6,7^, and the universal features of cellular senescence, that are cell cycle withdrawal^5^ and enhanced β-galactosidase activity at pH 6.0^9^, are shared with other cell states. Consequently, the precise identification of senescent cells based on their morpho-molecular traits remains a major obstacle to both mechanistic studies and therapeutic targeting.

Mitochondrial dysfunction has emerged as a key player in the onset of senescence across different models, with senescent cells showing increased mitochondrial biogenesis, defective turnover, and altered mitochondrial metabolism, namely mitochondrial coenzymes^8,10–12^. Despite the close association of mitochondrial homeostasis with lipid metabolism^13^, this is still an underexplored field, with few research studies that focus on understanding the lipid species involved^14^. Indeed, the identity, regulation, and function of lipids in senescence remain largely unknown, representing a major gap that hampers a full mechanistic understanding of this cellular state.

In the present study, we sought to fill this gap by comprehensively investigating metabolic alterations in senescent cancer cells in vitro through complementary technologies. Given the stressor- and cell type–dependent heterogeneity of the senescent phenotype, we established six cellular models—three human cancer cell lines treated with γ-rays or doxorubicin as senescence-inducing stimuli. Two-Photon Excitation Fluorescence (TPEF) microscopy was employed for label-free quantification of NAD(P)H and FAD fluorescence, providing insights into cell metabolism and mitochondrial organization at the single-cell level^15–17^. In parallel, matrix-Assisted Laser Desorption and Ionization (MALDI)-mass spectrometry was applied for the investigation of their lipid profiles. Mass spectrometry is considered a crucial tool for lipid research providing accurate and reliable information on lipid composition, profiling, and molecular identification^19,20^. Notably, senescent MCF7 cells have been shown to harbor a prominent subpopulation of aggressive, cannibalistic cells^30^, raising important questions about their functional heterogeneity and potential role in tumor progression. To gain deeper insight into the molecular features of this distinct senescent phenotype, we also performed RNA sequencing on both senescent and non-senescent MCF7 cells.

## Results

### Doxorubicin-induced and radiation-induced cancer senescent cells feature mitochondrial dysfunction

**Fig. 1:**
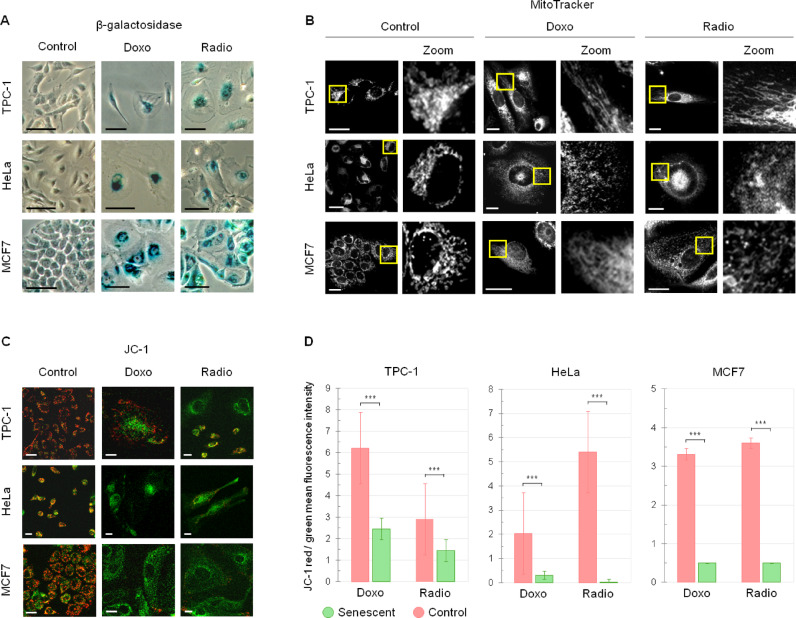
Senescent TPC-1, HeLa, and MCF7 feature mitochondrial damage. **(A)** Representative images of control and senescent (doxorubicin and radiation induced) TPC-1, HeLa and MCF7 cells stained for β-galactosidase activity (blue). Scale bar: 100 μm. **(B)** Representative images of control and senescent (doxorubicin and radiation induced) TPC-1, HeLa and MCF7 cells stained for mitochondria (with MitoTracker), with a magnification of a portion of each picture (Zoom) to better examine mitochondrial morphology. Scale bar: 50 μm. **(C)** Representative images of control and senescent (doxorubicin and radiation induced) TPC-1, HeLa and MCF7 cells marked with JC-1. JC-1 is a fluorescent dye that accumulates and dimerizes into active mitochondria, becoming red; when the mitochondrial membrane is depolarized, indicating mitochondrial damage, JC-1 stays in its monomeric form (green) in the cell cytoplasm instead. Scale bar: 50 μm. **(D)** Bar graphs showing the mean JC-1 derived red fluorescence intensity on green one for control and senescent (doxorubicin and radiation induced) senescent TPC-1, HeLa and MCF7 cells. For HeLa cells, 20 irradiated cells and 21 doxorubicin-treated cells were taken into consideration. For TPC-1 and MCF7 cells, 50 cells for each group were taken into consideration. T-tests were performed for statistical significance: ***p < 0.0005 (TPC-1 control vs. Radio: *p* = 3.0E-13; TPC-1 control vs. Doxo: *p* = 1.39E-20; HeLa control vs. Doxo: *p* = 0,000253; HeLa control vs. Radio: *p* = 1.40E-9; MCF7 control vs. Doxo: *p* = 1.00E-16; MCF7 control vs. Radio: *p* = 9.29E-21

Since the phenotype of cancer senescent cells can vary depending on the cell type and stimulus that triggered it^6,21,22^, for this study we employed different human cancer cells lines and senescence inducing agents. MCF7, HeLa and TPC-1 cells were treated with either doxorubicin or γ-irradiated. Both doxorubicin and γ-rays are commonly employed anti-cancer strategies and are widely known to induce senescence both in vitro and in vivo. Indeed, after 6 days of treatment, a great portion of cells was dead and the resistant ones displayed the enlarged, flat shape commonly associated with senescence^2,23^ (Fig. 1A). The establishment of senescence was corroborated through staining for β-galactosidase activity (Fig. 1A), a widely accepted and employed senescence biomarker. The positivity to X-gal staining (blue) was visibly higher in those cells that survived doxorubicin treatment or irradiation. As mitochondrial dysfunction has been shown to be one of the most common senescence features across several differentially induced senescent cells^8,10,12^, we set out to visualize the mitochondrial pattern and network in our models through staining with MitoTracker, a fluorescent dye that accumulates in mitochondria (Fig. 1B). As one can observe in Fig. 1B, mitochondria in control cells appear mostly as round spots, packed and regularly organized in space. This shape and pattern were lost in senescent cells. As mitochondria morphology is tightly linked with their functionality^11^, the apparent loss of order and reorganization of the mitochondrial network in senescent cells suggests mitochondrial damage and/or dysfunction. We further investigated mitochondrial dysfunction in our models by staining with JC-1 (Fig. 1C and D). As opposed to MitoTracker, with the JC-1 probe mitochondrial functionality is assessed based on the ratio between two different color fluorescent intensities in one’s sample instead of relying on one absolute intensity, accounting for more reliable results. As shown in Fig. 1C, senescent cells appear visibly greener and less red than their control counterparts, especially HeLa and MFC7. Indeed, the red-to-green mean fluorescent intensity ratio of all senescent cells was significantly lower than that of controls (Fig. 1D), indicating mitochondrial membrane depolarization in senescent cells. The authors precise that both JC-1 and MitoTracker derived fluorescence intensities in HeLa senescent cells were extremely low, possibly due to diminished dye penetration and/or retention, and this made it difficult to measure the fluorescent intensity derived from HeLa cells as accurately as for the other cell types. The results described in this section were included in Dr. Silvia Ghislanzoni’s PhD thesis *Investigation of Therapy-Induced Senescent Cancer Cell Phenotypes with Mass Spectrometry and Non-Linear Optical Microscopy* (10.21954/ou.ro.00101782*).*

### Detection of mitochondrial deregulation at the cellular level in unstained samples

**Fig. 2:**
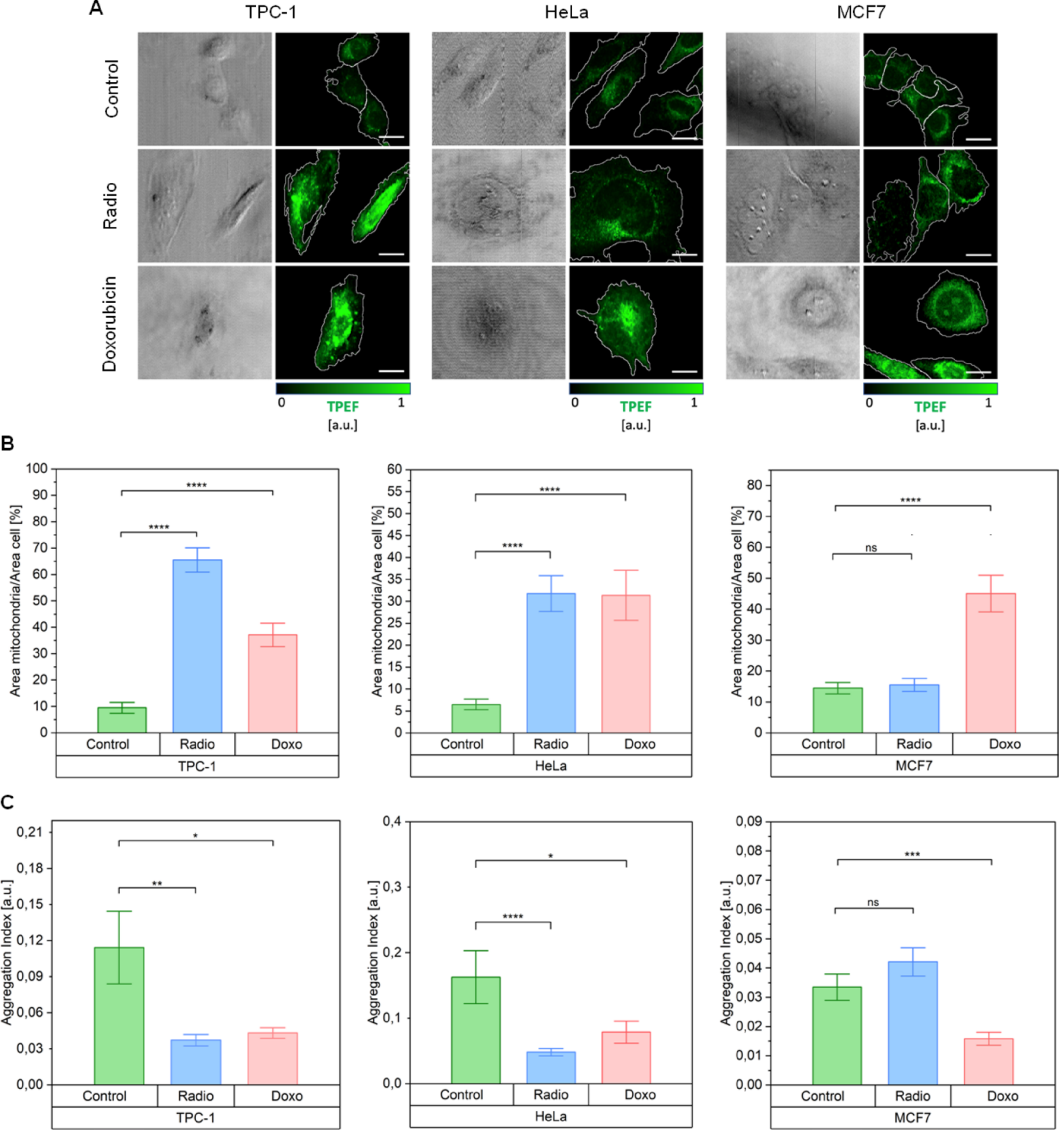
The metabolic alterations occurring in cells following the onset of the senescent state can be visualized in a completely label-free fashion. **(A)** Multimodal TPEF (green) images of control and senescent [radiation and doxorubicin induced) accompanied by the classical transmission image. Scale bar: 20 μm. **(B)** The percentage of cell area occupied by NAD(P)H and FAD coenzymes in the different cancer cell type measurements, compared by condition of the cells: control and senescent (radiation- and doxorubicin-induced). Two-sided Mann-Whitney U tests were performed for statistical significance: *0,01 < *p* ≤ 0,05; **p ≤ 0,01; ***p ≤ 0,001; ****p ≤ 0,0001. **(C)** The Aggregation Index results for NAD(P)H and FAD coenzymes in the different cancer cell lines measurements, compared by condition of the cells: control and senescent (radiation- and doxorubicin-induced). Two-sided Mann-Whitney U tests were performed for statistical significance: *0,01 < *p* ≤ 0,05; **p ≤ 0,01; ***p ≤ 0,001; ****p ≤ 0,0001. This Figure was included in Dr. Silvia Ghislanzoni’s PhD thesis *Investigation of Therapy-Induced Senescent Cancer Cell Phenotypes with Mass Spectrometry and Non-Linear Optical Microscopy* (10.21954/ou.ro.00101782*)*

Aiming at the visualization of metabolic alteration in senescent cells at the single cell level, we applied TPEF to control and senescent cells to measure the fluorescence signal derived from the metabolic cofactors NAD(P)H and FAD. Mitochondrial coenzymes are accepted biomarkers of metabolic states in different pathologies^18,24^, and we had already successfully performed this measurement in another cellular model of senescence in the past^25^. In Figs. 2A, the panels depict the acquired TPEF signal filtered below 600 nm. This signal comprises both NAD(P)H and FAD fluorescence, with the green-highlighted area denoting the region covered by the fluorescent signal. To quantitatively assess variations among distinct treatments for each cell type, we employed the Aggregation Index, defined as $\:\frac{Maximun\:TPFE\:signal\:[a.u.]}{[Area\:TPEF\:/Area\:Cell]\%}$, previously established^26^ and utilized in prior studies of ours with the same experimental setup^25^. This index relates the maximum density of mitochondrial coenzymes, scaling linearly with their endogenous fluorescence peak, with their distribution over the cell area^25^. Figure 2B showcases the cell area occupied by the TPFE signal normalized to the cell area for every cell line. NAD(P)H and FAD derived signals are more largely distributed in senescent cells with respect to their control counterparts. This result is consistent across all cell lines except for MCF7 senescent cells induced with γ-rays. Consistently, the aggregation index (Fig. 2C) proved to be significantly lower in all types of senescent cells than in their respective control counterparts (reflecting the cytosolic dispersal of mitochondrial coenzymes pinpointed by the TPEF/cell area metric and further indicating a decrease in local concentration of NAD(P)H and FADwith the exception of γ-rays triggered MCF7 ones. The results described in this section were included in Dr. Silvia Ghislanzoni’s PhD thesis *Investigation of Therapy-Induced Senescent Cancer Cell Phenotypes with Mass Spectrometry and Non-Linear Optical Microscopy* (10.21954/ou.ro.00101782*).*

### Lipidomic profiling reveals senescence-specific alterations across cell types

**Fig. 3:**
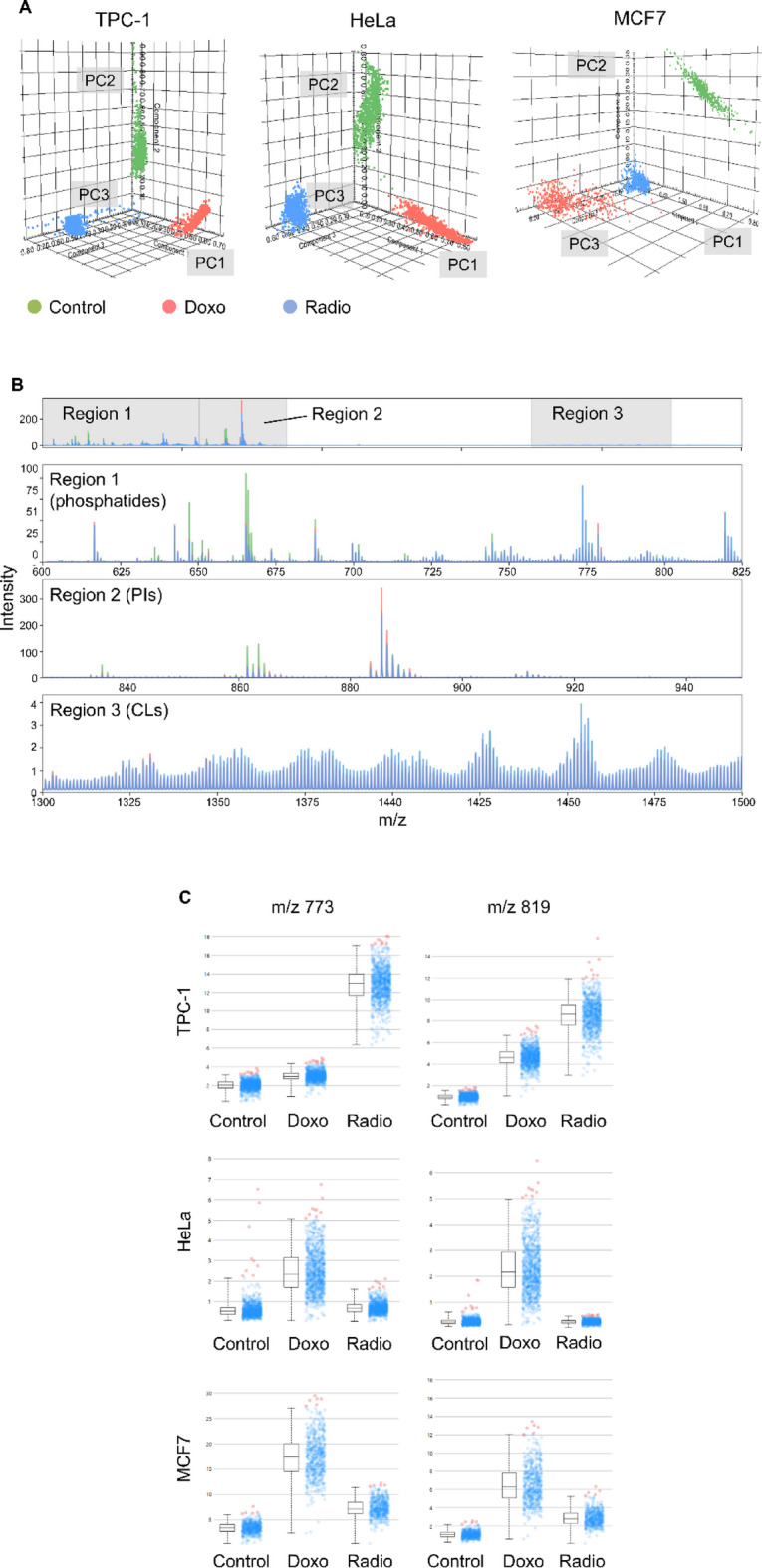
Exploration of the lipid profile with mass spectrometry. **(A)** Representative PLSA of control, doxorubicin induced, and radiation induced senescent TPC-1, HeLa, and MCF7 cells. **(B)** Representative mass spectrum of control (green), doxorubicin induced (red) and radiation induced (blue) senescent cells divided into three regions for the study of differential peaks in the three groups. The first region (Region 1), from m/z 600 to 825, includes peaks attributable mainly to mixed phosphatides; the second one (Region 2) ranges from 825 to 950 and is mostly occupied by PIs; the last region (Region 3), from 1300 to 1500, is where CLs are found. **(C)** Box plots for peaks 773 and 819 for TPC-1, HeLa, and MCF7 cells. The box plots presented in these figures are representative. Three biological replicates were analyzed for each group and cell line. For each experiment, hundreds of mass spectra were collected and averaged into a single spectrum. Specifically, the number of spectra used for each condition to generate these box plots was as follows: HeLa control, 812; HeLa Doxo, 894; HeLa radio, 797; TPC-1 control, 817; TPC-1 Doxo, 840; TPC-1 radio, 812; MCF7 control, 483; MCF7 Doxo, 527; and MCF7 radio, 562

For each cell line, control and senescent samples, induced either by doxorubicin or γ-irradiation, were collected and their organic fractions were extracted for mass spectrometry analysis. Three biological replicates per treatment group were analyzed, each comprising 300 to 900 mass spectra, which were averaged prior to statistical analysis. Probabilistic latent semantic analysis (pLSA) effectively discriminated senescent from control samples across all three cell types and further differentiated between doxorubicin- and radiation-induced senescence, suggesting distinct senescence-associated lipid signatures (Fig. 3). To facilitate analysis, spectra were divided into three m/z regions: Region 1 (m/z 600–825) predominantly including phosphatidylcholine (PC), phosphatidylethanolamine (PE), and sphingomyelin (SM) species; Region 2 (m/z 825–950) enriched in phosphatidylinositol (PI) species; and, Region 3 (m/z 1300–1500) associated with cardiolipins (CL), which are exclusive to mitochondrial membranes^27,28^. Differences between senescent and control cells were primarily observed as changes in peak intensity, rather than the presence or absence of specific lipid species. These intensity shifts varied depending on the cell type and senescence inducer. In TPC-1 cells, both forms of senescence were marked by increased intensity of a peak at m/z 616.47, corresponding to CerP (d16:1/18:0). While some lipid species decreased in doxorubicin-treated cells, they were elevated in radiation-induced senescence. Both stressors generally increased peak intensities in Region 1, with the exception of m/z 797.67 (SM d18:1/24:1), which decreased specifically in doxorubicin-treated cells. In Region 2, PI species were largely downregulated in senescent TPC-1 cells, although two PI peaks were elevated specifically under doxorubicin treatment. CLs (Region 3) were consistently increased in both senescence conditions, consistent with reports of increased mitochondrial mass during senescence^8,10^. In HeLa cells, doxorubicin-induced senescence was associated with a general increase in Regions 1 and 2 peak intensities, whereas radiation-induced senescence led mostly to decreased intensities, though some exceptions were observed. As with TPC-1, both senescence types in HeLa showed elevated CL levels, reinforcing the notion of mitochondrial involvement. Notably, two phosphatidylglycerol (PG) species, PG (18:1/18:1) at m/z 773 and PG (18:1/22:6) at m/z 819, were consistently upregulated across all senescent models, regardless of the inducing stressor^29^. Given that PGs are precursors of CLs, these findings support a broader pattern of mitochondrial membrane remodeling. Part of the results described in this section were included in Dr. Silvia Ghislanzoni’s PhD thesis *Investigation of Therapy-Induced Senescent Cancer Cell Phenotypes with Mass Spectrometry and Non-Linear Optical Microscopy* (10.21954/ou.ro.00101782*).*

### Gene expression profile of senescent-engulfed MCF-7 cells compared with those of senescent and control cells

**Fig. 4:**
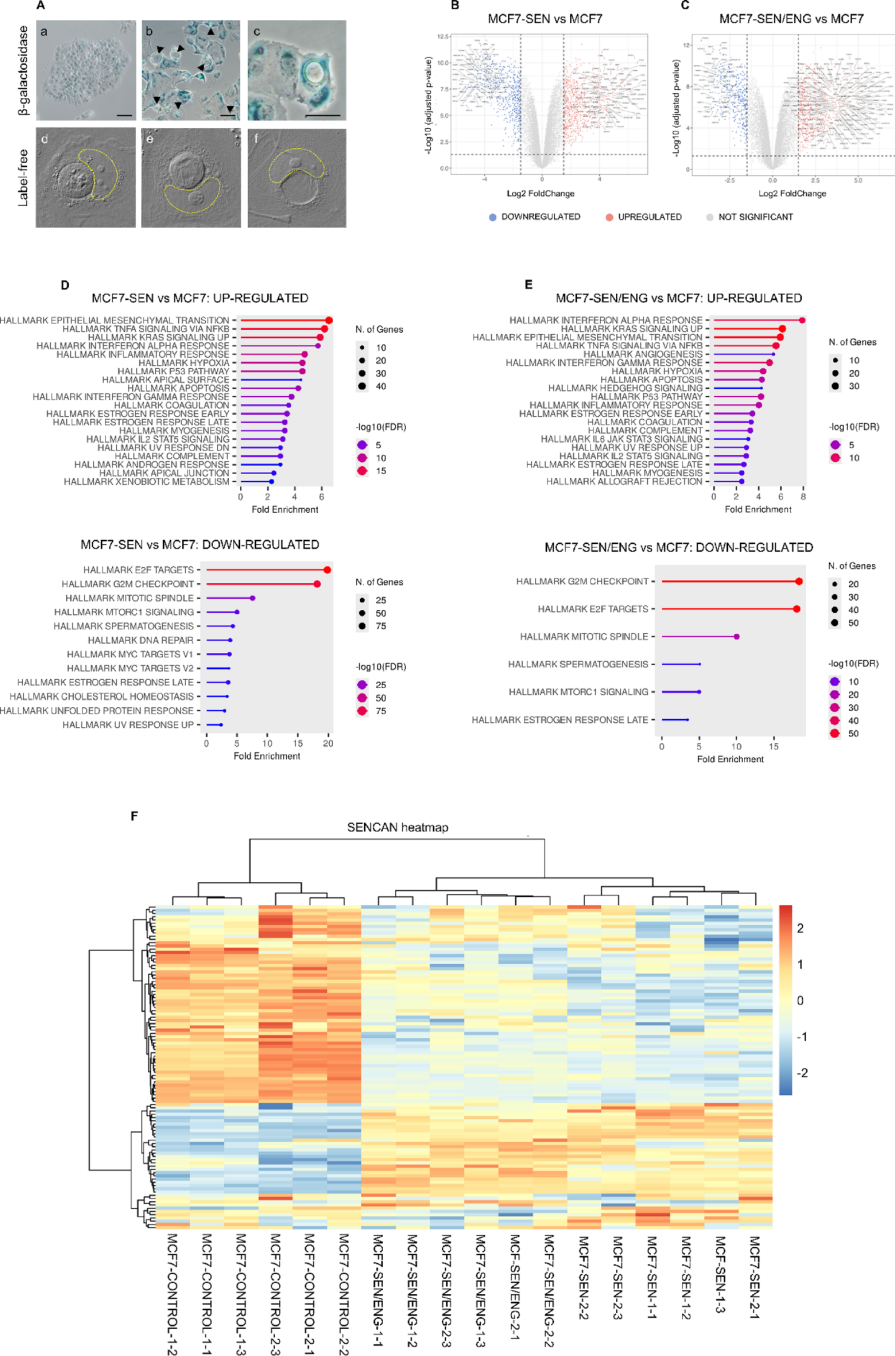
Investigation of MCF7 senescent engulfing cells. **A.** In the first row, control (a) and doxorubicin-treated (b) MCF7 cells stained for β-galactosidase activity (blue). In panel b, black arrows indicate cell-in-cell structures. In panel c, the detail of a cell-in-cell structure derived by the engulfing process. In the second row, images of unlabeled cell-in-cell structures (d and e) and of an engulfing cell with a large vacuole inside its cytoplasm (f). The nucleus of the engulfing cell is outlined in yellow. Figures in the second row were obtained through optical diffraction tomography at the Massachusetts Institute of Technology (Boston, MA, USA). **B** and **C.** Volcano plots illustrating the differentially expressed genes across each comparison: MCF7-SEN vs. MCF7 (**B**) and MCF7-SEN/ENG vs. MCF7 (**C**). Adjusted p value < 0.05 and |FC| > 1.5 were established as thresholds for significance. Genes that were not significantly up- or downregulated between groups are shown as well (grey dots). **D** and **E**. Hallmark.MSig.DB pathway analysis of significant up- and downregulated genes in MCF7-SEN (**D**) and MCF7-SEN/ENG (**E**) vs. MCF7 control samples using ShinyGO 0.82. **F.** SENCAN classifier applied to our RNA-seq dataset. The analysis was performed on untreated MCF7 cells (MCF7-CONTROL), MCF7 cells which survived 7 days of doxorubicin treatment (MCF7-DOXO), and a subpopulation of MCF7-SEN selected for the engulfing phenotype as described in the main text (MCF7-SEN/ENG)

Given the peculiar results of MCF7 cells, we set up to further explore this model. We had already noted that senescent MCF7 engulfed and degraded other cells in a massive vacuole within their cytoplasm^30^, a phenomenon previously described by us and others as well^31^. Briefly, when one cell becomes engulfed by another, distinctive round cell-in-cell structures can be observed (Fig. 4A). The internalized cells are progressively digested within large cytoplasmic vacuoles of the engulfing cells over the course of approximately 48 h. After the internalized cells are fully degraded, these vacuoles occupy the majority of the engulfing cell’s cytoplasm.

It is reasonable to hypothesize that engulfing cells would need a great amount of energy to engulf and degrade a whole cell, which could in part explain why the mitochondria of senescent MCF7 did not appear as dysfunctional as TPC-1 and HeLa (which did not engulf). To reach a deeper understanding of this model, including the engulfing subpopulation, we performed an RNA sequencing analysis on senescent MCF7 cells (MCF7-SEN) and senescent MCF7 cells enriched for the engulfing subpopulation (MCF7-SEN/ENG). MCF7-SEN and MCF7-SEN/ENG were compared with control MCF7 (MCF7). Using an adjusted p value < 0.05 and |FC| > 1.5 as a threshold for significance, we found 1066 and 1384 differentially expressed genes (DEGs among the three groups (Fig. 4 and Supplementary Files 2, 3 and 4).

To further corroborate the establishment of the senescent state in our models, we applied the SENCAN classifier to the RNA-seq dataset (Fig. 4F), together with single-sample GSEA (ssGSEA) scoring of senescence-associated gene sets (Supplementary File 5). Both MCF7-SEN and MCF7-SEN/ENG samples exhibited high SENCAN senescence scores, consistent with the transcriptional repression of cell-cycle–related programs and the activation of canonical senescence pathways.

The transition from control MCF7 cells to the senescent MCF7-SEN MCF7-SEN/ENG phenotypes was predominantly characterized by gene overexpression. In MCF7-SEN cells, genes showed altered expression, with 791 genes (57%) upregulated and 593 genes (43%) downregulated. In contrast, in MCF7-SEN/ENG cells, the majority of differentially expressed genes (675 out of 1066; 63%) were upregulated, while 391 genes (37%) were downregulated.

We analyzed differentially expressed genes (DEGs) among MCF7-SEN, MCF7-SEN/ENG vs. control MCF7 cells by applying various bioinformatics tools to bulk RNA-seq data. Gene Set Enrichment Analysis (GSEA) was performed using the 50 Hallmark gene sets from the Molecular Signatures Database (MSigDB), which represent well-defined and curated biological processes [**33**].

This analysis revealed several interrelated pathways commonly associated with cellular senescence. In MCF7-SEN cells, we observed significant enrichment of multiple pathways associated with cellular senescence and stress responses. Among the most enriched gene sets were Epithelial-Mesenchymal Transition (EMT), TNFA Signaling via NFKB, KRAS Signaling UP, Interferon Alpha and Gamma Responses, Inflammatory Response, Hypoxia, P53 Pathway, and Apoptosis. Additional enriched pathways included Estrogen Response (Early and Late), Coagulation, IL2-STAT5 Signaling, and Xenobiotic Metabolism, reflecting the activation of immune signaling, metabolic changes, and hormonal responses typically associated with the senescent state.

In MCF7-SEN/ENG cells, we identified a partially overlapping but distinct set of upregulated gene sets. Notably, Interferon Alpha Response, KRAS Signaling UP, EMT, TNFA Signaling via NFKB, Angiogenesis, and Hedgehog Signaling were among the top enriched pathways. The presence of Allograft Rejection and IL6-JAK-STAT3 Signaling suggests enhanced immune-related activity and pro-inflammatory signaling in engulfed senescent cells. While many stress-related pathways were shared with MCF7-SEN, some pathways such as Xenobiotic Metabolism and Cholesterol Homeostasis showed differential regulation between the two senescent models. Overall, these results indicate that while both senescent phenotypes exhibit a core transcriptional program involving inflammation, immune activation, and epithelial remodeling, the engulfed MCF7-SEN/ENG cells display additional features that may reflect enhanced or sustained senescence signaling.

Regarding mitochondrial-related functions, hallmark sets such as MTORC1 signaling were downregulated in both senescent phenotypes reflecting a reduced anabolic activity or impaired mitochondrial biogenesis. Taken together, the mitochondrial hallmark alterations observed in both MCF7‑SEN and MCF7‑SEN/ENG models underscore a multifaceted mitochondrial stress phenotype including an increased ROS and hypoxia signaling, suppressed proteostasis and mitophagy, metabolic reprogramming, and pro-apoptotic polarization.

**Fig. 5:**
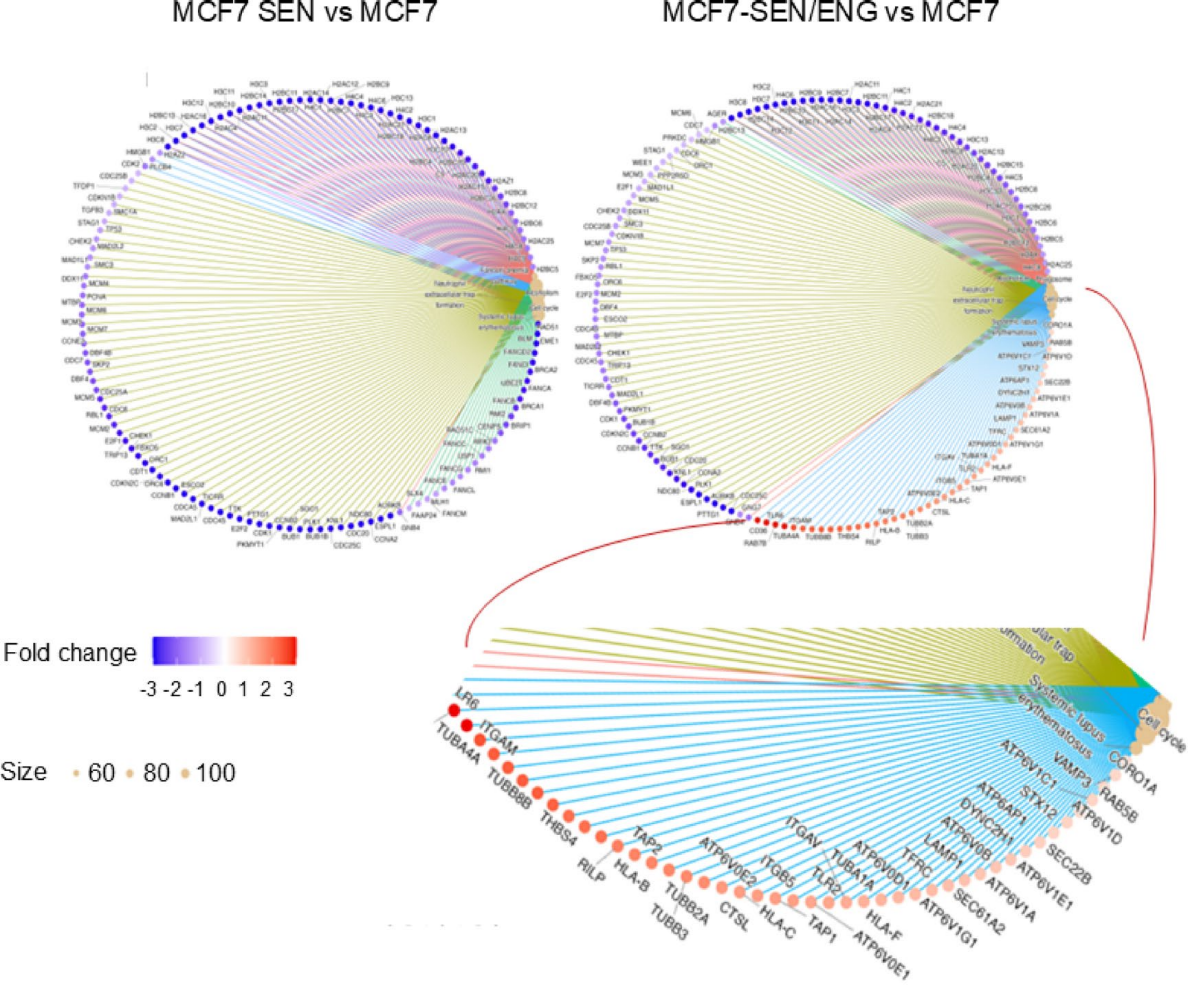
Differentially expressed genes in MCF7-SEN compared to control cells and MCF7-SEN/ENG compared to control cells using KEGG. Cnetplots indicate the expression and distribution of upregulated (in orange) and downregulated genes (in blue) genes. The blue triangle delineates upregulated genes in the MCF7-SEN/ENG group; among this, of particular interest for this work: V-ATPase subunits (ATP6V0 and ATP6V1); phagosome-related and lysosomal genes (RAB5B, VAMP3, STX12, TFRC, LAMP1, and SEC61A2); innate immune and phagocytic surface receptors (TLR6, ITGAM (CD11b), CD36, and CORO1A); cell cycle and cytoskeleton-related proteins (CDC25C, TUBB-type tubulins, THBS4 genes). KEGG (https://urldefense.com/v3/__http://www.kegg.jp/__;!!CF15FET90Tp8!CrhwGCo8SxjU7ManMNz1EQOBJpez--Ygr5rvc3uOJx1GYIru2rc1O8Yx_ZeATA6STge6v8QSzJGvJ3s-hMnjykvmdbC-leYLbOCWsqdtAu0$ or https://urldefense.com/v3/__http://www.genome.jp/kegg__;!!CF15FET90Tp8!CrhwGCo8SxjU7ManMNz1EQOBJpez--Ygr5rvc3uOJx1GYIru2rc1O8Yx_ZeATA6STge6v8QSzJGvJ3s-hMnjykvmdbC-leYLbOCW0OMCPvU$) was used to determine the main metabolic pathways and signaling pathways implicating differentially expressed genes^62–64^.(permission number 253251). A total of 69 genes in the metabolics pathway (KEGG_hsa01100) had P-values < 0.05, but the top candidate ESCC genes were MTAP, GAPDH, DCTD, POLD2 and AMDHD1

DEGs were identified in the comparisons MCF7-SEN vs. MCF7, MCF7-SEN/ENG vs. MCF7, and MCF7-SEN/ENG vs. MCF7-SEN, and subjected to functional enrichment analysis using KEGG database (p-value < 0.05) (Fig. 5). Upregulated V‑ATPase components likely enable higher lysosomal acidification and autophagic/phagocytic flux. Trafficking and lysosomal genes support enhanced vesicle fusion and cargo processing. Surface receptors tied to phagocytosis reinforce the hypothesis of a cannibalistic phenotype in senescent MCF7 cells. Cytoskeletal and secretory regulators suggest structural and functional changes linked to the SASP.

Together, these gene expression changes describe MCF7-SEN/ENG as cells undergoing a metabolic adaptation with mitochondrial stress phenotype and lysosomal activation, potentially explaining a cannibalistic behavior, probably used as a survival strategy under conditions of extreme energy depletion^32^.

## Discussion

In this study, we employed TPEF microscopy, MALDI mass spectrometry, and RNA sequencing to explore different aspects of the metabolic reprogramming that occurs upon the establishment of senescence in human cancer cell lines. While mitochondrial dysfunction is a recognized hallmark of senescence, it has been mainly explored in replicative models^8,10^. Here, we confirm that therapy-induced senescent cells also display mitochondrial alterations, including membrane depolarization and redistribution of NAD(P)H and FAD, detected both by classical staining and label-free imaging. Beyond this confirmation, the novel contribution of this work lies in providing a multi-level metabolic characterization of therapy induced senescence that integrates optical, lipidomic, and transcriptomic analyses.

NLO analyses revealed a significant increase in the autofluorescence signal of mitochondrial cofactors in all senescent cells examined, except for radiation-induced MCF7 cells. Since these cofactors accumulate in mitochondria^33^, this observation suggests an increase in mitochondrial biomass, a hallmark of senescence. It is also known that mitochondrial dysfunction can lead to NADH leakage into the cytoplasm^33^, which may contribute to the widespread TPEF signal observed in senescent cells. Notably, senescent TPC-1 and HeLa cells showed an increased signal of cardiolipin precursors compared to controls. In senescent MCF7 cells, we did not observe consistent accumulation of mitochondrial coenzymes or cardiolipins precursors. However, peaks at m/z 773 and 819 were detected, corresponding to PG (18:1/18:1) and PG (18:1/22:6), respectively^29^. Since phosphatidylglycerols (PGs) are precursors of cardiolipins^34, 35^, their overexpression may indicate deregulation of mitochondrial function and dynamics. In a model of doxorubicin-induced senescence, mitochondrial lipid remodeling was reported, involving changes in PGs and PIs and an increase in membrane fluidity^34^. This aspect could be particularly relevant for engulfing senescent cells, which must undergo extensive membrane deformation to internalize neighboring cells. These results expand our prior observations on early therapy-induced senescence by providing a quantitative, multi-omic description of mitochondrial lipid remodeling and redox alterations.

While β-galactosidase staining and morphological features were used to define the senescent state, we acknowledge that inclusion of additional canonical senescence markers, as well as assessment of SASP components, would further strengthen the phenotypic characterization. Nonetheless, the combination of morphological changes, stable cell-cycle arrest, and mitochondrial dysfunction provides robust and widely accepted evidence of a senescent phenotype consistent with prior literature. RNA-seq analyses of MCF7 senescent and engulfing-senescent cells further identified a transcriptional program consistent with mitochondrial stress, inflammatory activation, and metabolic rewiring. In addition, to further support the senescence phenotype in our models, we performed the SENCAN classifier and ssGSEA analysis (Supplementary File 5) on our RNA seq. dataset, providing further evidence of the establishment of the senescent state. Both MCF7-SEN and MCF7-SEN/ENG groups exhibited features commonly associated with the senescent state^36,37^, such as strong inhibition of cell cycle progression, increased activation of the DNA damage response (DDR), and upregulation of estrogen response signaling. Additionally, both models showed robust enrichment in hallmark pathways such as EMT, TNFα signaling via NF-κB, KRAS signaling, interferon-α and -γ responses, and inflammatory response^38,39^. Altogether, these findings reflect a mitochondrial stress phenotype characterized by the accumulation of reactive oxygen species (ROS), impaired oxygen homeostasis, and activation of intrinsic apoptotic pathways. Notably, our work also revealed new features of senescent engulfing cells, including upregulation of phagocytosis-related, lysosomal, and lipid-trafficking genes. These pathways likely support a nutrient recycling mechanism that compensates for energy depletion. This behavior distinguishes the engulfing subpopulation as a novel metabolic variant of therapy-induced senescence, not previously characterized in terms of metabolism in our earlier optical studies. This is consistent with the observed downregulation of mTORC1 and mitochondrial UPR signaling in the MCF7-SEN/ENG group. In fact, we observed consistent downregulation of mTORC1 and mitochondrial UPR pathways in this subpupolation. A suppressed mTORC1 signature may reflect reduced anabolic activity or impaired mitochondrial biogenesis, consistent with literature describing mTORC1 inhibition as a lifespan-extending intervention^40^. Concurrently, reduced UPR likely indicates diminished mitochondrial proteostasis capacity, as often seen in cancer^41^. Enrichment of glycolytic gene signatures supports a metabolic shift from oxidative phosphorylation to glycolysis, consistent with the metabolic rewiring characteristic of senescence, possibly due to impaired OXPHOS function and ROS elevation.

MCF7-SEN/ENG cells also showed activation of phagocytosis-related and hypoxia-related gene signatures. These may support nutrient recovery, as also suggested by the overexpression of CD36, a receptor that cooperates with integrins and TLRs to promote phagocytosis^42,43^. CD36 is also involved in the trafficking of lipids to lysosomes, potentially contributing to lysosomal calcium dysregulation and dysfunction^44,45^. In macrophages, the phagocytosis of apoptotic cells via CD36 provides lipids, amino acids, and nucleotides that are degraded in lysosomes and activate the mTORC1 pathway^46,47^, supporting repair and anti-inflammatory responses^48^. In our senescent cells, we observed a reduction in mTORC1 activity suggesting that CD36 may help compensate for low mTORC1 signaling by supporting nutrient uptake or, under overload conditions, contribute to lysosomal stress.

Nutrient deprivation can arise not only from lack of external nutrients but also from intracellular shortages of essential molecules. Under such conditions, the uptake of apoptotic cells can provide metabolic support for survival and mTORC1 reactivation. Recently, Ratto et al.^49^. showed that cells can acquire exogenous amino acids via endocytosis and lysosomal degradation of extracellular proteins. In amino acid-rich environments, this nutritional use of extracellular proteins is blocked by the mTORC1 sensor through a yet unclear mechanism^50^. However, in low mTORC1 activity states, the V1 domains of V-ATPases assemble on lysosomes to form active proton pumps, lowering the pH and increasing proteolytic activity. This represents a rapid adaptation mechanism to nutrient availability, mobilizing the latent catabolic potential of lysosomes. In our previous study on MCF7-SEN engulfing cells^30^, we discussed how engulfment may be followed by acidic lysosomal degradation^31,51^. Su et al.^52^. showed that vacuolar acidification induces death of engulfed cells in various breast cancer models, describing vacuoles as “giant lysosomes”. These findings suggest that amino acids released from vacuolar degradation may activate mTORC1, potentially controlling vacuolar maturation and division^47,51^. While mTORC1 suppression is a known correlate of reduced anabolic activity, our data show that it occurs in parallel with enhanced lipid uptake and degradation, suggesting a previously unrecognized balance between catabolic adaptation and metabolic stress in therapy-induced senescence.

One limitation of this study is the absence of direct functional assays of mitochondrial activity, such as Seahorse flux analysis, oxygen consumption rate, ATP quantification, or ROS measurement. Although strongly supported by multiple complementary approaches, our conclusions are based on indirect evidence of mitochondrial activity rather than direct functional measurements. Nevertheless, several mitochondrial hallmarks observed across our models, including JC-1 membrane depolarization, altered NAD(P)H and FAD distribution, and enrichment of oxidative stress–related transcriptional pathways, strongly support the presence of elevated ROS and oxidative stress. This interpretation is consistent with multiple studies linking mitochondrial depolarization and NAD(P)H redistribution with ROS accumulation in senescent cells^8,10^. Future studies combining these complementary bioenergetic assays with multimodal imaging will be essential to validate the metabolic traits identified here and to further dissect the energetic consequences of therapy-induced senescence.

Despite its limitations, our work confirms the central role of mitochondrial dysfunction in senescence but extends the field by demonstrating that cancer therapy-induced senescence encompasses metabolically distinct sub-states. In particular, we identified a lipid-driven remodeling of mitochondrial and plasma membranes that may enable engulfment and survival under nutrient limitation. This integrated multi-omic framework provides new insight into the metabolic plasticity of senescent cancer cells and points to membrane dynamics and lipid metabolism as potential therapeutic vulnerabilities.

## Methods

All experiments were performed using at least two independent biological replicates.

### Cell lines and cultures

All cell lines were purchased from ATCC (USA). Human breast adenocarcinoma MCF7 cells were maintained in RPMI 1640 Medium (Gibco) supplemented with 10% fetal bovine serum (FBS, Gibco), while human cervical adenocarcinoma HeLa cells and human papillary thyroid carcinoma TPC-1 cells were maintained in Dulbecco’s modified Eagle’s Medium (DMEM, Gibco) supplemented with 10% FBS and 1% glutamine (Gibco). These specific cell lines were selected as models for therapy-induced senescence because they are well characterized, widely used cancer cell lines from distinct tissue origins, each with different genetic backgrounds that influence senescence pathways. MCF-7 (ER-positive breast carcinoma) retains functional p53/Rb and is known to undergo senescence in response to chemotherapeutics or irradiation. HeLa carries HPV E6/E7 and enables exploration of senescence in the context of disrupted p53/Rb control. TPC-1 (papillary thyroid carcinoma with RET/PTC1 rearrangement) offers an oncogene-driven context relevant to thyroid cancer. Their established characterization and translational relevance provide a robust panel to explore the metabolic alterations arising with the establishment of cancer therapy induced senescence.

### Treatments

To obtain the senescent phenotype, adherent cells were treated with 250 nM doxorubicin (Doxo, Sigma), or irradiated at 10 Gy released by γ-rays from 137Cs sources of IBL147 biological irradiator (0.65 Gy = min). Cells were observed and/or analyzed from 6 to 8 days after treatment. Control (untreated) cells, whose proliferation was not arrested by treatment, were split when necessary to avoid apoptosis due to overgrowth until observation (typically once after seeding and before the end point), or alternatively collected prior to full confluence, typically 72–96 h after seeding. To obtain a MCF7 population enriched in engulfing cells, MCF7 cells were treated with doxorubicin 24 h after seeding as described above. After treatment with doxorubicin, surviving MCF7 cells consistently engulfed and degraded neighboring ones, with engulfing cells constituted about 30% of the total cell population. To increase the proportion of engulfing cells, MCF7 cells were collected through trypsinization and re-seeded after 6 days of treatment. The complete engulfing process is described in our work *Optical Diffraction Tomography and Raman Confocal Microscopy for the Investigation of Vacuoles Associated with Cancer Senescent Engulfing Cells*^30^. Briefly, the internalization of one cell into another originates distinctive, round cell-in-cell structures. The internalized cells were gradually digested within large cytoplasmic vacuoles of the engulfing cells over approximately 48 h. Following the degradation of the internalized cells, these vacuoles occupied most of the engulfing cell cytoplasm. The vacuoles persisted for about 12–15 h before progressively shrinking and disappearing within the next 15 h, after which the previously engulfing cells regained a normal morphology and were indistinguishable from neighboring non-engulfing ones.

### β-galactosidase staining

Cells were fixed and stained according to the instructions of SA-β-gal Staining Kit (Cell Signaling), then observed under a light microscope (Leica DM IRB microscope, Leica Microsystems).

### Mitotracker staining

Mitochondria were stained in live cells with 100 nM MitoTracker Deep Red FM (Invitrogen, Italy) diluted in the culture medium: cells were incubated with the probe at 37 °C and 5% CO_2_ for 30 min; after the incubation period, cells were gently washed with PBS and either fixed with 4% PFA for 10 min to be observed later or observed directly with the confocal microscope Leica TCS SP8 X (Leica Microsystems GmbH, Germany).

### JC-1 staining

Mitochondrial membrane potential was investigated using the JC-1 dye (Thermo Fisher, Italy). The dye was diluted to a final concentration of 0.1 µg/mL in the culture medium. Control, irradiated and doxorubicin-treated cells were incubated with the probe at 37 °C and 5% CO_2_ for 15 min. Then, cells were gently washed with PBS and imaged immediately in PBS with the confocal microscope Leica TCS SP8 X (Leica Microsystems GmbH, Germany). JC-1 exhibits a potential-dependent accumulation in mitochondria, indicated by a fluorescence emission shift from green to red: polarized mitochondria are marked by punctate red fluorescent staining. If mitochondria are depolarized, the red punctate staining is replaced by diffuse green fluorescence. The samples were excited at wavelengths 498 and 568 nm. Green and red fluorescent intensities were measured using the software ImageJ. A t-test was performed to compare mean red-to-green fluorescence intensity ratios between control and treated cells.

### Mass spectrometry

Mass spectrometry analyses were performed with a Bruker UltrafleXtreme MALDI TOF mass spectrometer (Germany) operating in reflectron mode and equipped with a frequency tripled 355 nm Nd: YAG laser at repetition rates up to 10 kHz. Control and senescent cells were trypsinized, pelleted and washed with PBS. The pellet of each sample was resuspended in 700 µL of tris–sucrose buffer and centrifuged for 10 min at 4000 r.p.m. The supernatant was discharged. The extraction of lipids from the cells was performed using the method of *Bligh and Dye*^53^. 1 µL of lipid suspension was deposed on Idium Tin Oxide (ITO) slides and covered with the matrix 9-AA. A TM-sprayer nebulizer (HTX Technologies) was used to apply the matrix for MALDI-MSI analysis. The 9-AA matrix (Sigma-Aldrich) was prepared in 2-propanol/ACN (60/40, v/v) to a final concentration of 10 mg/mL. For its application, the temperature of the nozzle was set to 80 °C, the pressure 10 psi, and the LC pump flow rate 0.12 mL/min. After matrix application, samples were left in the desiccator until MALDI-MSI spectra acquisition. Samples were analyzed in negative-ion mode using a Bruker UltrafleXtreme MALDI TOF mass spectrometer operating in reflectron mode and equipped with a frequency-tripled 355 nm Nd: YAG laser at repetition rates up to 10 kHz. Laser power and laser focus position were manually fine-tuned before each acquisition to ensure optimal data quality and comparable signal intensity. Imaging experiments were controlled by FlexImaging 4.1 software (Bruker Daltonics, MA, USA) with a raster size of 50 μm. At each raster position, 200 laser shots were summed to generate a representative spectrum for each pixel with the digitizer sampling rate at 1.25 GS/s. Spectra were acquired in the *m/z* 800–1600 range at each pixel position. For each experiment, 300 to 900 mass spectra were collected and averaged into one; the number of mass spectra collected depended on the size of the area occupied by the organic extract on the ITO slide. The data from the analysis of control and senescent cells were co-registered to the acquired MALDI-MSI data and normalized to total ion count (TIC) and visualized by FlexImaging. SCiLS lab version 2022 Pro (SCiLS GmbH, Germany) was used for data analysis. Briefly, MALDI-MSI reduced datasets were imported into SCiLS Lab, converted to the SCiLS SL format, and then normalized to the TIC. SCiLS was used to obtain the spatial distribution and create box and dot plots of normalized signal peak intensities. For the identification of lipid species, tandem mass spectrometry (MS/MS) analysis was performed using a laser ionization fragmentation technology (LIFT) approach as described by *Suckau et al.*^54^. Characteristic product ions of precursor ions in the MS/MS spectrum were used to identify the lipid class by searching accurate MS/MS fragmentation pattern data in LIPID MAPS (Lipidomics Gateway, http://www.lipidmaps.org/). To compensate for the relatively low resolving power of approximately 5000 at *m/z* 885.56, tentative assignments were made only if the proposed lipid species were previously described in the literature.

### Two-Photon excitation fluorescence

Cells were cultured and treated on 22 × 22 × 0.17 mm quartz slides. At the desired endpoint, untreated (control) and senescent (treated) cells were gently washed with PBS and fixed with 4% PFA for 10 min. Each quartz slide was mounted, upside-down, in the middle of a clean, 50 × 22 × 0.17 mm one (with the cells placed between the two slides) and the four hedges of each smaller slide sealed to the bigger ones using nail polish. We used a homebuilt multimodal optical microscope featuring seven different NLO modalities including two-photon excited fluorescence (TPEF and E-TPEF), along with linear transmission light. This setup has been utilized and described in detail in a previous study of ours^25^. For image analysis, images were processed via the Fiji-ImageJ software. Both dark and bright 1-pixel outliers in every channel of multimodal images were median-filtered to correct the extreme pixel values given by cosmic rays. An automated circular shift of image columns is used to compensate for distortion effects due to the serpentine-like motion of the motorized sample stage. A universal threshold of 0.25 arbitrary unit (a.u.) was set to distinguish the endogenous fluorescence signal from the diffused background signal that presented outside of cells. To calculate the maximum value of TPEF displayed by cells in an image; in order to quantify the maximum coenzymes density within cells, we considered the average value of the 10% highest pixel values. This procedure, together with the aforementioned median filtering of outlier pixels, allowed us to avoid spurious TPEF pixel values and obtain a metric that effectively scales with the maximum density of fluorescent scatterers in the FOV. To quantify treatment-induced mitochondrial re-arrangements accounting for NAD(P)H and FAD density, we calculated the mitochondrial aggregation index, defined as $\:\frac{Maximun\:TPFE\:signal\:[a.u.]}{[Area\:TPEF\:/Area\:Cell]\%}$. This index, previously established^26^ and utilized in prior studies of ours with the same experimental setup^25^, is used to quantify the local concentration of mitochondrial coenzymes, as it relates the maximum abundance of mitochondrial coenzymes, scaling linearly with their intrinsic fluorescence intensity, normalized to their spatial distribution across the cell area. As the aggregation index should feature at the numerator a value scaling with the density of target molecules regardless of their spatial distribution, which is considered in the denominator instead, we used TPEF maxima rather than mean or standard deviation values.

### RNA sequencing analysis

Total RNA was isolated from MCF-7 cells treated with either vehicle control (0.1% DMSO) or 250 nM doxorubicin for 72 h using the RNeasy Mini Kit (Qiagen, Santa Clarita, CA) on an automated QIAcube extractor (Qiagen, Santa Clarita, CA), following the manufacturer’s instructions.

The RNA quantity was measured using Qubit 4 (Agilent Technologies, Palo Alto, CA), and RNA integrity was assessed with the TapeStation 4200 (Agilent Technologies, Santa Clara, CA). Data on RNA concentrations and quality are provided in Supplementary File 1.

All samples met the quality criteria and were processed for RNA sequencing. RNA-seq libraries were prepared using the Illumina Stranded Total RNA Prep, Ligation with Ribo-Zero™ Plus kit (Illumina, San Diego, CA, USA, Cat# 20040525), following the manufacturer’s protocol.

Briefly, libraries were generated from 100 ng of total RNA, which was initially depleted of abundant ribosomal RNA (rRNA). The remaining RNA was then reverse transcribed into cDNA, followed by ligation and amplification steps to add adapters for clustering and sequencing. The final library concentrations were measured using Qubit 4, and quality control was performed with the TapeStation 4200.

All libraries were purified and diluted to a starting concentration of 0.5 nM. The pooled libraries were sequenced at 2 × 75 base pairs on the Illumina NovaSeq 6000 platform (Illumina, San Diego, CA, USA) using a NovaSeq 6000 SP Reagent Kit v1.5 (200 cycles).

Quality data for the sequencing libraries are provided in Supplementary File 1. The raw FASTQ files have been submitted to GEO under the accession number GSE298654.

Raw gene count matrix is provided as Supplementary File 1. RNA-seq reads were mapped to the reference human genome (hg38) using the STAR aligner^55^ and the count read numbers per gene was performed with htseq-count^56^. To analyze the gene expression profile, we applied the SamR pipeline^57^ within R software (version 4.4.2). Briefly, the dataset of raw counts was first filtered to remove genes with low reads using the function filterByExpr by EdgeR package and then normalized using the trimmed mean of M-values (TMM)^58^. This method estimates a scale factor used to reduce technical bias between samples that result from differences in library size. We then applied the counts-per-million (CPM) transformation and log2-transformed the data for downstream analysis. The RNA-seq adaptation of the Significance Analysis of Microarrays (SAM) method implemented in *samr* package was used to rank genes based on their expression patterns across conditions, generating a ranking metric that was subsequently used for enrichment analysis.

To assess the senescence state of our samples, we applied the SENCAN classifier to TMM-normalized logCPM expression values. This gene set (see Supplementary File 6) encompasses canonical senescence markers (e.g., *CDKN1A*, *HMGB1*), components of mRNA processing and splicing (*SNRPA*, *SRSF2*), interferon-responsive genes (*IFIT2*, *ISG15*), histone variants (*H4C8*, *H2AC20*), and the telomere maintenance factor *RTEL1*. The SENCAN classifier was originally developed by training machine-learning models on 13 cancer cell lines driven into senescence by alisertib or etoposide, with the aim of defining shared molecular features and vulnerabilities of therapy-induced senescence. This model enables classification of the senescent state in cancer-derived transcriptomic samples and was used here as an orthogonal validation of the senescence phenotype.

To identify specific biological pathways enriched in the selected feature genes, functional annotation exploiting Gene Set Enrichment Analysis (GSEA), Kyoto Encyclopedia of Genes and Genomes (KEGG), Gene Ontology (GO) and Reactome datasets were performed through the *clusterProfiler*^59^, *ReactomePA*^60^ and *GSEABase*^61^ R packages. In particular, KEGG (https://urldefense.com/v3/__http://www.kegg.jp/__;!!CF15FET90Tp8!CrhwGCo8SxjU7ManMNz1EQOBJpez--Ygr5rvc3uOJx1GYIru2rc1O8Yx_ZeATA6STge6v8QSzJGvJ3s-hMnjykvmdbC-leYLbOCWsqdtAu0$ or https://urldefense.com/v3/__http://www.genome.jp/kegg__;!!CF15FET90Tp8!CrhwGCo8SxjU7ManMNz1EQOBJpez--Ygr5rvc3uOJx1GYIru2rc1O8Yx_ZeATA6STge6v8QSzJGvJ3s-hMnjykvmdbC-leYLbOCW0OMCPvU$) was used to determine the main metabolic pathways and signaling pathways implicating differentially expressed genes^62–64^ (permission number 253251). The enrichment was assessed using a rank-based approach, where enrichment scores (ES) were computed, and their statistical significance was determined through permutation testing. The resulting p-values were adjusted for multiple testing using the Benjamini-Hochberg procedure, and pathways were considered significantly enriched if the adjusted p-value was ≤ 0.05, with a minimum of 15 genes per pathway.

## Supplementary Information

Below is the link to the electronic supplementary material.

Supplementary Material 1

Supplementary Material 2

Supplementary Material 3

## Data Availability

The RNAseq datasets generated and/or analysed during the current study are available in the GEO repository with public release date: 2025-12-10 (https://www.ncbi.nlm.nih.gov/geo/query/acc.cgi?acc=GSE298654).
