# Supplementary material for: Metabolic profiling of therapy-induced senescent cancer cells via TPEF, MALDI-MS, and RNA-sequencing
Source: Sci Rep. 2025 Dec 17;16:2678. doi: 10.1038/s41598-025-32573-y (PMC12823619; doi:10.1038/s41598-025-32573-y)
Supplement: Supplementary file 3 — Supplementary Material 3 [file 41598_2025_32573_MOESM3_ESM.pdf]

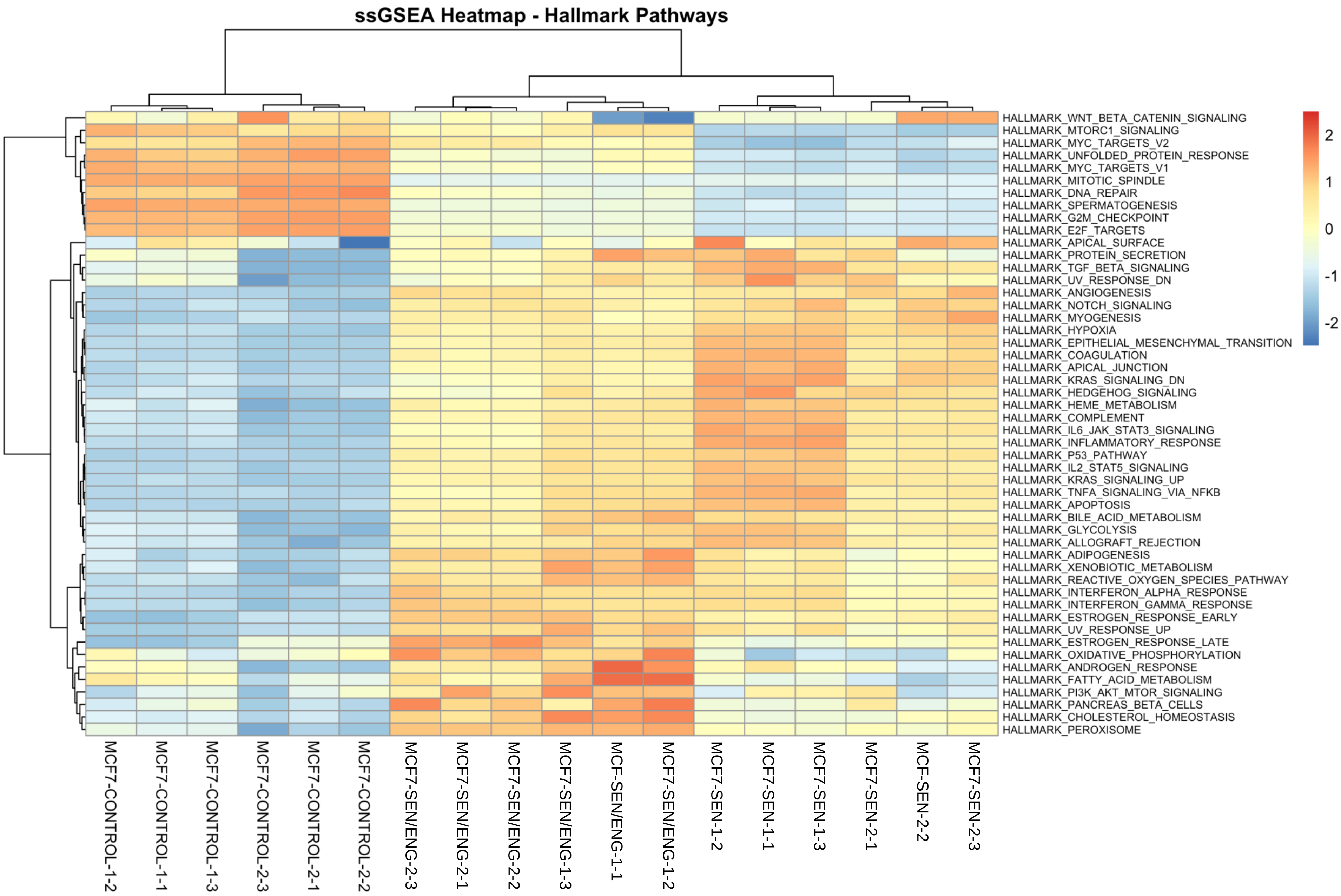

Supplementary File 5. Single-sample Gene Set Enrichment Analysis performed on untreated MCF7 cells (MCF7-CONTROL), MCF7 cells which survived 7 days of doxorubicin treatment (MCF7-DOXO), and a subpopulation of MCF7-SEN selected for the engulfing phenotype as described in the main text (MCF7-SEN/ENG).
